# Influence of First-Line Antibiotics on the Antibacterial Activities of Acetone Stem Bark Extract of *Acacia mearnsii* De Wild. against Drug-Resistant Bacterial Isolates

**DOI:** 10.1155/2014/423751

**Published:** 2014-07-01

**Authors:** Olufunmiso O. Olajuyigbe, Roger M. Coopoosamy

**Affiliations:** Department of Nature Conservation, Mangosuthu University of Technology, P.O. Box 12363, Jacobs, KwaZulu-Natal, Durban 4026, South Africa

## Abstract

*Background.* This study was aimed at evaluating the antibacterial activity of the acetone extract of *A. mearnsii* and its interactions with antibiotics against some resistant bacterial strains. *Methods.* The antibacterial susceptibility testing was determined by agar diffusion and macrobroth dilution methods while the checkerboard method was used for the determination of synergy between the antibiotics and the extract. *Results.* The results showed that the susceptibility of the different bacterial isolates was concentration dependent for the extract and the different antibiotics. With the exception of *S. marcescens*, the inhibition zones of the extract produced by 20 mg/mL ranged between 18 and 32 mm. While metronidazole did not inhibit any of the bacterial isolates, all the antibiotics and their combinations, except for ciprofloxacin and its combination, did not inhibit *Enterococcus faecalis*. The antibacterial combinations were more of being antagonistic than of being synergistic in the agar diffusion assay. From the macrobroth dilution, the extract and the antibiotics exerted a varied degree of inhibitory effect on the test organisms. The MIC values of the acetone extract which are in mg/mL are lower than those of the different antibiotics which are in *μ*g/mL. From the checkerboard assay, the antibacterial combinations showed varied degrees of interactions including synergism, additive, indifference, and antagonism interactions. While antagonistic and additive interactions were 14.44%, indifference interaction was 22.22% and synergistic interaction was 37.78% of the antibacterial combinations against the test isolates. While the additivity/indifference interactions indicated no interactions, the antagonistic interaction may be considered as a negative interaction that could result in toxicity and suboptimal bioactivity. *Conclusion.* The synergistic effects of the herbal-drug combinations may be harnessed for the discovery and development of more rational evidence-based drug combinations with optimized efficiency in the prevention of multidrug resistance and therapy of multifactorial diseases.

## 1. Introduction

Antibiotics play an important role in preventing and treating diseases. However, antibiotic resistance has become a global public health problem due to its excessive use which has resulted in many emerging multidrug-resistant microorganisms. The indiscriminate use of these antimicrobial drugs and bacterial genetic ability to transmit and acquire resistance to drugs utilized as therapeutic agents has further compromised the use of newer generations of antibiotics [1]. As a consequence, antibiotic-resistant bacteria are continuously emerging and becoming more problematic in the medical field [2] with infectious diseases, mediated by drug-resistant pathogens, being associated with increased morbidity, mortality, health care costs, and longer hospital stays [3].

Although infectious diseases, a worldwide concern, remain one of the world's leading causes of premature death [4] and a major cause of debility [5], the significant effects of multidrug resistance in the same vein are synonymous due to infectious diseases [6]. Multidrug-resistant bacteria present an emerging threat worldwide in hospitalized children and adult patients [7]. While about 45 percent of isolates from patients in South Africa are resistant to penicillin, erythromycin, ampicillin, clindamycin, tetracycline, and sulphonamides due to the quick adaptation of bacteria to new environmental conditions [8] and antibiotic-resistant enterococci and metallo-*β*-lactamase-producing Enterobacteriaceae have become major global causes of nosocomial infections [9] due to prior therapy [10] and inappropriate prescriptions [11]. The concomitant resistance of these bacteria to more than three different antimicrobial classes has limited treatment options [12, 13]. With the growing microbial resistance to conventional antimicrobial agents, the development of novel and alternative therapeutic agents with activity against such resistant strains has become necessary.

Consequently, natural plant-derived antimicrobials, having a long history of providing the much needed novel therapeutics [14], proving to be an invaluable source of medicine for humans [15], are largely utilized as crude extracts in the form of herbal remedies. However, owing to the emergence of new infectious diseases and the increases in multidrug-resistant bacteria, one of the employable strategies to overcome these challenges is the combination of antimicrobial agents such herbal medicines and the conventional antibiotics. While drug interactions are possible when herbs are taken concurrently with drugs, Tasneem [16] indicated that the herbal medicines can inhibit, exaggerate, or negate the actions of a prescription drug. Betoni et al. [17] and Adwan et al. [18] reported that these combinations can enhance the efficacy of the antimicrobial agents and be an alternative to treat infections caused by multidrug-resistant microorganisms not susceptible to any effective therapy readily available. While Cupp [19] adduced that drug interactions could be caused by impurities, Kobilinsky et al. [20] showed that two or more compounds interact in ways that mutually enhance, amplify, or potentiate each other's effect more significantly than the simple sum of the effects of each agent involved.

*Acacia mearnsii* De Wild. (Fabaceae) is a member of the genus Acacia. In South Africa, where it was introduced to over 150 years ago primarily for tanning industry [21], it is considered a wild and a notorious plant because of its ability to compete with indigenous plants and populate a large expanse of land sporadically. Although there has been a dearth of scientific reports indicating its pharmacological importance, Olajuyigbe and Afolayan [22–24] reported that* A. mearnsii* is a medicinal plant of ethnobotanical and pharmacological importance. To further establish its pharmacological relevance in an era when there is a need for effective therapy against infections with multidrug-resistant bacteria, this study was aimed at evaluating the antibacterial activity of the acetone extract of* A. mearnsii* alone, first-line antibiotics alone, and the effects of their antibacterial combinations against some resistant bacterial strains.

## 2. Materials and Methods

### 2.1. Collection of Plant Material and Extract Preparation

Stem bark of* Acacia mearnsii* De Wild. was collected from the plant growing in Nkonkobe municipality, Eastern Cape, South Africa. The bark sample was air-dried at room temperature and pulverized using a milling machine. The extract of the bark material was prepared according to Basri and Fan [25] description. About 100 g of the pulverized sample was extracted with 500 mL of methanol for 48 h with shaking (Stuart Scientific Orbital Shaker, UK). The extract was filtered through Whatman number 1 filter paper and concentrated under reduced pressure at 40°C using a rotary evaporator (Laborota 4000 efficient, Heidolph, Germany). The extract was redissolved in 25% v/v acetone before being made up to the required concentrations for bioassay with the sterile distilled water. The reconstituted extract solution was sterilized by filtering through 0.45 *μ*m membrane filter and tested for sterility after membrane filtration by introducing 1 mL of the extract into 9 mL of sterile nutrient broth before being incubated at 37°C for 24 h. A sterile extract was indicated by the absence of turbidity in the broth after the incubation period.

### 2.2. Source of Bacterial Strains and Preparation of Bacterial Inocula Preparation

The bacteria used in this study included* Escherichia coli* ATCC 25922,* Bacillus cereus* ATCC 10702,* Pseudomonas aeruginosa* ATCC 19582,* Serratia *marcescens ATCC 9986,* Enterococcus faecalis* KZN,* Staphylococcus aureus*_OK1_,* Shigella flexneri* KZN,* Micrococcus luteus*,* Proteus vulgaris* CSIR 0030, and* Salmonella typhi* ATCC 13311. The antibacterial assays were carried out using Mueller-Hinton II Agar (Biolab) and broth. The inocula of the test bacteria were prepared using the colony suspension method [26]. Colonies picked from 24 h old cultures grown on nutrient agar were used to make suspensions of the test organisms in saline solution to give an optical density of approximately 0.1 at 600 nm. The suspension was then diluted 1 : 100 by transferring 0.1 mL of the bacterial suspension to 9.9 mL of sterile nutrient broth before being used.

### 2.3. Antibiotics Used in This Study

Antibiotic powders of amoxicillin, ciprofloxacin, chloramphenicol, erythromycin, kanamycin, metronidazole, nalidixic acid, and tetracycline were used. Stock antibiotic solutions were prepared and dilutions made according to the Clinical Laboratory Standardization Institute (CLSI) method or manufacturer's recommendations [27].

### 2.4. Antibiotic Susceptibility Testing Using Agar Diffusion Method

Each of the isolates was standardized using colony suspension method. Each strain's suspension was matched with 0.5 McFarland standards to give a resultant concentration of 1.0 × 10^8^ cfu/mL. The antibiotic susceptibility testing was determined using the modified Kirby-Bauer diffusion technique [28] by swabbing the Mueller-Hinton agar (MHA) (Oxoids, UK) plates with the resultant saline suspension of each strain. Wells were then bored into the agar medium with heat sterilized 6 mm cork borer. The wells were filled with 100 *μ*L of different concentrations prepared for the methanolic extract alone, antibiotics alone, and their combinations taking care not to allow spillage of the solutions onto the surface of the agar. The plates were allowed to stand for at least 30 min before being incubated at 37°C for 24 h [29]. The determinations were done in duplicate. The acetone concentration in the starting well was <2.5% v/v and its effects alone on the growth of bacteria was examined and effects were seen. After 24 h of incubation, the plates were examined for zones of inhibition. The diameter of the zones of inhibition produced by the extract alone, antibiotic alone, and their combinations were measured and interpreted using the CLSI zone diameter interpretative standards [30].

### 2.5. Determination of Minimal Inhibitory Concentration (MIC)

The susceptibility of the selected bacteria to the antimicrobial agents and their minimum inhibitory concentrations (MIC) were determined in duplicate by the standard macrobroth dilution method in Mueller-Hinton broth [31]. To determine the MIC of each antibiotic, the concentrations used for each of the antibiotics (0.0019–500) *μ*g/mL and those of extract (0.0012–5) mg/mL were prepared by serial dilution in Mueller-Hinton broth. To determine their combinatorial effects, different concentrations of each of the antibiotics and the extract were combined. The tubes were inoculated with 100 *μ*L of each of the bacterial strains. Macrodilution tubes containing 2.5% acetone was inoculated and the turbidity following incubation was compared with a control without 2.5% acetone. The turbidity showed that 2.5% of acetone has no inhibitory effects on the bacterial isolates. Blank Mueller-Hinton broth was used as negative control. The bacterial containing tubes were incubated aerobically at 37°C for 24 h. Each combination assay was performed two times. The MIC was defined as the lowest antibiotic or acetone extract concentration which prevented visible growth [32].

### 2.6. Checkerboard Assay

The checkerboard method, commonly used for measuring interactive inhibitions [33], was used for the determination of synergy between the antibiotics and the acetone extract. The range of drug concentration used in the checkerboard assay was such that the dilution range encompassed the MIC for each drug used in the analysis. The fractional inhibitory concentration (FIC) was derived from the lowest concentrations of the extract and the antibiotics in combination permitting no visible growth of the test organisms in the Mueller-Hinton broth after incubation for 24 h at 37°C [34]. FIC indices were calculated using the formula: FIC index = (MIC of extract in combination/MIC of extract alone) + (MIC of antibiotics in combination/MIC of antibiotics alone). In antimicrobial combination, Schelz et al. [35] defined synergy as *∑*FIC ≤ 0.5, additivity as 0.5 < *∑*FIC ≤ 1, indifference as 1 < *∑*FIC ≤ 4, and antagonism as *∑*FIC > 4.

## 3. Results

This study showed that the bacterial susceptibility was concentration dependent for the extract and the different antibiotics used even though they exhibited varied antibacterial activities. With the exception of* S. marcescens*, the inhibition zones at the least concentration of the extract, 5 mg/mL, were between 13 and 25 mm while those of the highest concentration of 20 mg/mL ranged between 18 and 32 mm. While metronidazole did not inhibit any of bacterial isolates, except* E. coli*, erythromycin alone did not inhibit* E. coli*,* S. marcescens,* and* S. typhi*, all the antibiotics and their combinations, except for ciprofloxacin and its combination, did not inhibit* E. faecalis*. Though all the bacterial isolates were susceptible to the combination of metronidazole and the extract and* S. typhi* was susceptible to the combination of erythromycin and the extract, all the isolates were susceptible to ciprofloxacin and its combination with the extract while* E. coli*,* B. cereus*,* Ps. aeruginosa*,* M. luteus, P. vulgaris,* and* S. flexneri* were susceptible to tetracycline, amoxicillin, nalidixic acid, chloramphenicol, and kanamycin and their combinations with the extract.* S. typhi* was susceptible to all the antibacterial agents except metronidazole and their combinations while* S. marcescens* was susceptible to all antibacterial agents and their combinations with the exception of erythromycin and its combination.* S. aureus* was susceptible to the antibacterial agents, except nalidixic acid, and their combinations. A consideration for the degree of antibacterial activities in term of the sizes of the inhibition zones, at the highest concentration of the individual antibacterial agent and their combination, showed that the average inhibition zones for the extract ranged between 18 and 32 mm, erythromycin alone between 0 and 34 mm, its combination between 0 and 28 mm, tetracycline alone between 0 and 36 mm, its combination between 0 and 38 mm, metronidazole alone 0, its combination between 16 and 23 mm, amoxicillin alone between 0 and 36 mm, its combination between 0 and 40 mm, ciprofloxacin alone between 27 and 40 mm, its combination between 25 and 35 mm, nalidixic acid between 0 and 34 mm, its combination between 0 and 30 mm, chloramphenicol between 0 and 35, its combination between 0 and 30 mm, kanamycin between 0 and 32 mm, and its combination between 0 and 34 mm. Considering susceptibility of all the isolates against each antibiotic at the highest concentration, nalidixic acid and kanamycin were the most active against* S. typhi*, amoxicillin and chloramphenicol were the most active against* M. luteus*, ciprofloxacin was most active against* S. marcescens* and* S. typhi*, chloramphenicol was most active against* M. luteus* and* S. typhi*, metronidazole was most active against* E. coli*, tetracycline was most active against* S. aureus*_OK1,_ and erythromycin was most active against* Ps. aeruginosa* of each antibiotic. A comparison of the antibacterial activity of each antibiotic and its combination showed that the antibacterial combinations were more of being antagonistic than of being synergistic in the agar diffusion assay. With the exception of metronidazole having its combination showing synergy in comparison to its no antibacterial effect when used alone and the combination of tetracycline having most of its combination as being synergistic, most of the antibacterial combinations with other antibiotics were mostly antagonistic (Table 1).

Although the antibacterial combinations in agar diffusion assay were mostly antagonistic interactions, the macrobroth dilution assay showed the degree of the antibacterial activities of the antibiotics alone and their combinations with the acetone extract of the* A. mearnsii.* From the macrobroth dilution, the acetone extract and the antibiotics exerted a varied degree of inhibitory effect on the test organisms. The MIC values ranged between 0.039 and 0.625 mg/mL for the extract, 0.048 and 12.5 *μ*g/mL for erythromycin, between 0.244 and 31.25 *μ*g/mL for tetracycline, between 15.625 and 62.5 *μ*g/mL for metronidazole, between 0.244 and 250 *μ*g/mL for amoxicillin, between 0.039 and 1.25 *μ*g/mL for ciprofloxacin, between 1.953 and 62.5 *μ*g/mL for nalidixic acid, between 0.977 and 31.25 *μ*g/mL for chloramphenicol, and between 1.953 and 500.0 *μ*g/mL for kanamycin. The MIC values of the acetone extract which are in mg/mL are lower than those of the different antibiotics which are in *μ*g/mL. According to the MIC breakpoints recommended by BSAC [29], strains of Enterobacteriaceae,* Staphylococcus* species,* Enterococcus* species, and Gram positive aerobes with MIC values of ≤8 mg/L for chloramphenicol, ≤0.5 mg/L for ciprofloxacin, ≤1 mg/L for tetracycline, ≤0.25 mg/L for erythromycin, ≤4 mg/L for amoxicillin, ≤8 mg/L for kanamycin, ≤4 mg/L for metronidazole, and ≤16 mg/L for nalidixic acid are classified as being susceptible. If the MIC breakpoints for the antibiotics are considered and the susceptibility results are interpreted according to BSAC [29], the MIC breakpoint showed that only* M. luteus* was resistant to ciprofloxacin,* B. cereus*,* S. marcescens,* and* S. flexneri* were resistant to amoxicillin,* E. faecalis* KZN,* S. aureus*_OK1_, and* P. vulgaris* CSIR 0030 were resistant to chloramphenicol,* E. faecalis* KZN,* S. aureus*_OK1_,* P. vulgaris* CSIR 0030,* M. luteus,* and* S. flexneri* KZN were resistant to nalidixic acid,* E. coli*,* S. marcescens*,* E. faecalis*,* S. typhi,* and* M. luteus* were resistant to erythromycin, and* S. marcescens*,* E. faecalis,* and* M. luteus* were resistant to tetracycline. With the exception of* S. marcescens* which was susceptible to kanamycin at a concentration of 1.953 *μ*g/mL, all the isolates were resistant to metronidazole. There is a correlation between the antibacterial activities of the extract and the antibiotics in agar diffusion and macrobroth dilution assays (Table 2).

The* in vitro* antibacterial activity of these antibiotics and their combinations was further assessed with the checkerboard assay to determine the fractional inhibitory concentration (FIC) index. When the antibacterial combination resulting in synergy as *∑*FIC ≤ 0.5, additivity as 0.5 < *∑*FIC ≤ 1, indifference as 1 < *∑*FIC ≤ 4, and antagonism as *∑*FIC > 4 were considered, the antibacterial combinations showed varied degree of interactions including synergism, additive, indifference, and antagonism interactions. While the extract had highest additive interaction with chloramphenicol and highest indifference interaction with nalidixic acid, antagonistic interaction was more recorded with metronidazole. The combination of the extract and tetracycline showed the highest synergistic interaction, followed by the extract's combination with ciprofloxacin and amoxicillin which is greater than those of erythromycin. While chloramphenicol and amoxicillin had no antagonistic interaction against any bacterial isolates, chloramphenicol and kanamycin showed synergy greater than those of nalidixic acid and metronidazole. While antagonistic and additive interactions were 14.44%, indifference interaction was 22.22% and synergistic interaction was 37.78% of the antibacterial combinations against the test bacterial isolates. While the fractional inhibitory concentration indices (FICI) for the synergistic interaction was between 0.062 and 0.50, the FICI for the additive was between 0.509 and 1.0, that of indifference was between 1.062 and 3.0, and that of synergistic interaction was between 4.13 and 18.01 (Table 3).

## 4. Discussion

Stepping up researches in phytomedicine, the study of herb-drug interaction, focusing on synergy and finding scientific rationale for the therapeutic superiority of many herbal drug extracts derived from traditional medicine as compared with single constituents thereof [36], has gained much attention since the mid-1990s [37]. This is in addition to promote the ethnopharmacological importance of herbal medicines, justifying their applicability in folkloric medicines and determining their potentials as sources of novel therapeutic agents which has become essential to study medicinal plants with folklore reputations intensively [38]. While the concurrent uses of pharmaceuticals with herbal remedies are rarely declared by majority of patients to medical practitioners [39] and there is little evidence to guide clinicians and consumers on interactions between natural plant products and medicines [40], investigating herbal-drug interactions against multidrug resistant bacteria becomes a major reason for the development of improved strategies for the management of microbial infections.

In this study, the inhibition zones produced by the extract ranging between 18 and 32 mm at the highest concentration of 20 mg/mL and those of all the antibiotics, with the exception of those not showing susceptibility at all, fell within the range of susceptible and resistant limits between 16 and 23 mm set by the BSAC [29] and are in agreement with range of diameter of inhibition zones earlier reported by Ogbeche et al. [41] and Akinyemi et al. [42]. Also, the determination of the minimum inhibitory concentrations (MIC) and the subsequent determination of the fractional inhibitory concentrations of the antibacterial combinations are quantitative methods based on the principle that test organisms had contact with the serially diluted antimicrobial agents and their combinations. While MIC determinations are widely used and accepted for measuring the degree of microbial susceptibility to inhibitors [43], the varied degree of susceptibility exhibited by the test bacterial isolates may be attributed to the intrinsic levels of tolerance to antimicrobials in the tested bacteria. While extracts with MIC ≤ 1 mg/mL are considered having high antibacterial activities [44] and phytochemicals are classified antimicrobials when susceptibility tests had MIC between 0.1 and 1 mg/mL [45], having MIC ranging between 0.039 and 0.625 mg/mL showed that the acetone extract had significant antibacterial activities. However, there exist variations between the activities of the extract and the antibiotics. These variations may be due to the mixtures of bioactive compounds present in the extract compared to the pure compounds contained in the antibiotics.

Although investigating drug combinations can give valuable insights into the significance of synergistic and antagonistic interactions of dissimilar drugs [46], the concomitant administration of herbal medicine and prescribed drugs is an unidentified challenge in the treatment of bacterial infection as drug-herbal interactions may occur. From this study, varied degree of different interactions including synergism, additivity, indifference, and antagonism were recorded. The synergistic interactions between the acetone extract and the different antibiotics are in agreement with previous studies that showed that crude extract of plants possess the ability to enhance the activity of antimicrobial agents [47, 48]. The synergistic interaction resulted in the significant reduction of the MIC and an increase in the antibacterial activities of the antibiotics.

Although the principal mechanism of action of antibiotics includes interference with cell wall synthesis, inhibition of protein synthesis, interference with nucleic acid synthesis, and inhibition of a metabolic pathway [49], *β*-Lactam agents inhibit synthesis of the bacterial cell wall by interfering with the enzymes needed for the synthesis of the peptidoglycan layer [50]. Macrolides, aminoglycosides, tetracyclines, and chloramphenicol produce their antibacterial effects by inhibiting protein synthesis [49, 50]. Fluoroquinolones exert their antibacterial effects by disrupting DNA synthesis and causing lethal double-strand DNA breaks during DNA replication [51]. Polymyxins increase bacterial membrane permeability and cause leakage of bacterial contents [52]. The cyclic lipopeptide daptomycin apparently inserts its lipid tail into the bacterial cell membrane [53] to cause membrane depolarization and eventual death of the bacterium. On the contrary, while scientific validation of the antimicrobial properties of plants has been extensively reported [54] and natural plant-derived products may give a new source of antimicrobial agents with possibly novel mechanisms of action [55, 56], little information is available on their mechanisms of action in bacteria.

However, while Tomlinson and Palombo [57] reported that extract of the leaves of* Eremophila duttonii* distorted the integrity of the cytoplasmic membrane of* S. aureus* to cause increased membrane permeability, Ultee et al. [58] and Lambert et al. [59] indicated membrane damage, changes in intracellular pH, membrane potential, and adenosine triphosphate (ATP) synthesis. Kris-Etherton et al. [60], Manson [61], and Surh [62] reported interference with some metabolic processes and modulation of gene expression and signal transduction pathways. Chapple et al. [63] and Lohner and Blondelle [64] reported that the extract could have promoted a local disturbance and the alteration of the physicochemical properties of the outer membrane, the membrane proteins and porin pathways to cause an increase in membrane permeability, and the inflow of the antibacterial agents. Even though the mechanisms of synergy in combined antibacterial agents are speculative, it may be due to a combination of effects instead of a single effect, decreased aggressiveness of the bacterial isolates and increased concentration of antibacterial components at the target sites [65], synergistic multitarget effects [66], and antagonization of resistance mechanisms of the bacteria [67]. With the synergy recorded in this study, the natural antimicrobials may have facilitated the penetration of each of the antibiotics through the outer layers of the bacterial cell wall, blocked the inhibitory effects of protective enzymes, or interfered with single or multiple metabolic targets of the antibiotic [67, 68]. The pharmacologically active complex compounds that could have been formed between the extract and each antibiotic may, probably, have allowed sufficient amount of each of the antibacterial agents to adsorb, diffuse, penetrate, and interact with the target sites thereby preventing the active mechanism of resistance in the isolates.

Considering the additivity/indifference interactions, however, Meletiadis et al. [69] indicated that most* in vitro* combination studies resulting in FIC indices within the range of 0.5 and 4.0 are of no interactions. As a result, concurrent administration of this extract and the antibiotics to which it has produced additivity/indifference interaction could result in either the extract or the antibiotics acting individually in a monotherapeutic manner while possibly attacking the same or different target sites in pathogens simultaneously and offer alternative therapy to infections caused by the multidrug-resistant bacteria. However, the antagonistic interaction may be considered as a negative interaction that could result in toxicity and suboptimal bioactivity. Hence, combining acetone extract of* A. mearnsii* with the antibiotics to which it showed antagonism may not yield a positive therapeutic effect or lead to adverse herbal-drug interactions.

In conclusion, the emergence of multidrug-resistant bacteria has seriously reduced the number of empirical agents suitable for selected indications. While multidrug strategy is based on the fact that many diseases have a multicausal etiology and a complex pathophysiology, many infectious diseases can be treated more effectively with pharmaceutical combinations than with a single antibacterial agent. From this study, the extract had a significant antibacterial activity and exhibited synergy, additive, indifference, and antagonistic effects in combination with the different antibiotics. While the synergistic effects may have overcome the intrinsic resistance in the bacterial strains and the additive/indifference effects may imply that the extract and the antibiotics acted individually in each bacterial strain to achieve effective antibacterial activity, the antagonistic effects indicated that the extract may not be combined with some antibiotics for therapeutic purposes. The synergistic effects of the herbal-drug combinations may, however, be harnessed and assessed for the discovery and development of more rational evidence-based drug combinations with optimized efficiency in the prevention of multidrug resistance and therapy of multifactorial diseases.

## Figures and Tables

**Table 1:** Average inhibition zones (±1.0 mm) produced by acetone extract of *Acacia mearnsii* and their different combinations.

	AMA alone			Ery alone			Combined AMA + Ery			Tet alone			Combined AMA + Tet		
	5	10	20	62.5	125	250	1.25 + 62.5	2.5 + 125	5 + 250	62.5	125	250	1.25 + 62.5	2.5 + 125	5 + 250
	mg/mL			*µ*g/mL			mg/mL + *µ*g/mL			*µ*g/mL			mg/mL + *µ*g/mL		
*Escherichia coli* ATCC 25922	13	15	18	0	0	0	0	0	0	22	24	27	22	25	28
*Bacillus cereus* ATCC 10702	17	20	23	25	28	30	20	23	27	25	27	31	25	28	32
*Pseudomonas aeruginosa* ATCC 19582	16	18	21	26	30	34	21	23	25	22	25	28	25	27	30
*Serratia marcescens* ATCC 9986	0	14	18	0	0	0	0	0	0	16	18	22	15	18	22
*Enterococcus faecalis* KZN	17	20	23	0	0	0	0	0	0	0	0	0	0	0	0
*Staphylococcus aureus*_ OK1_	17	19	21	28	30	33	25	26	28	31	34	36	30	34	38
*Salmonella typhi* ATCC 13311	25	29	32	0	0	0	0	14	16	29	31	33	31	33	36
*Micrococcus luteus *	14	16	19	20	23	27	17	20	24	22	26	30	27	30	33
*Proteus vulgaris* CSIR 0030	18	21	23	22	23	25	22	24	25	23	26	28	25	27	30
*Shigella flexneri* KZN	20	22	24	25	27	30	15	27	31	22	23	25	25	26	28

	AMA alone			Met alone			Combined AMA + Met			Amx alone			Combine AMA + Amx		
	5	10	20	62.5	125	250	1.25 + 62.5	2.5 + 125	5 + 250	62.5	125	250	1.25 + 62.5	2.5 + 125	5 + 250
	mg/mL			*µ*g/mL			mg/mL + *µ*g/mL			*µ*g/mL			mg/mL + *µ*g/mL		

*Escherichia coli* ATCC 25922	13	15	18	0	15	20	0	15	17	22	24	27	22	24	27
*Bacillus cereus* ATCC 10702	17	20	23	0	0	0	17	20	22	15	18	20	18	20	22
*Pseudomonas aeruginosa* ATCC 19582	16	18	21	0	0	0	14	17	19	20	21	22	19	20	22
*Serratia marcescens* ATCC 9986	0	14	18	0	0	0	0	14	18	14	17	20	17	20	23
*Enterococcus faecalis* KZN	17	20	23	0	0	0	17	20	23	0	0	0	0	0	0
*Staphylococcus aureus*_ OK1_	17	19	21	0	0	0	16	21	24	28	30	32	27	29	32
*Salmonella typhi* ATCC 13311	25	29	32	0	0	0	0	15	18	28	32	34	30	35	37
*Micrococcus luteus *	14	16	19	0	0	0	11	14	16	28	32	36	33	36	40
*Proteus vulgaris* CSIR 0030	18	21	23	0	0	0	14	16	18	18	20	22	20	23	2
*Shigella flexneri* KZN	20	22	24	0	0	0	15	18	20	19	20	23	22	25	26

	AMA alone			Cip alone			Combined AMA + Cip			Nal alone			Combined AMA + Nal		
	5	10	20	1.25	2.5	5	1.25 + 1.25	2.5 + 2.5	5 + 5	62.5	125	250	1.25 + 62.5	2.5 + 125	5 + 250
	mg/mL			*µ*g/mL			mg/mL + *µ*g/mL			*µ*g/mL			mg/mL + *µ*g/mL		

*Escherichia coli* ATCC 25922	13	15	18	30	33	35	20	24	27	25	27	30	20	24	28
*Bacillus cereus* ATCC 10702	17	20	23	20	24	27	18	22	27	20	25	28	17	19	22
*Pseudomonas aeruginosa* ATCC 19582	16	18	21	21	25	28	18	21	25	24	27	30	17	19	24
*Serratia marcescens* ATCC 9986	0	14	18	32	35	40	26	29	32	27	30	33	25	28	30
*Enterococcus faecalis* KZN	17	20	23	33	38	42	21	24	28	0	0	0	0	0	0
*Staphylococcus aureus*_ OK1_	17	19	21	30	35	38	25	27	30	0	0	0	15	18	21
*Salmonella typhi* ATCC 13311	25	29	32	32	37	40	15	16	18	29	32	34	24	26	29
*Micrococcus luteus *	14	16	19	23	28	32	19	24	27	0	0	0	0	0	0
*Proteus vulgaris* CSIR 0030	18	21	23	25	26	28	23	25	27	24	26	30	23	25	29
*Shigella flexneri* KZN	20	22	24	28	30	33	30	32	35	18	20	22	20	21	23

	AMA alone			Chl alone			Combined AMA + Chl			Kan alone			Combined AMA + Kan		
	5	10	20	62.5	125	250	1.25 + 62.5	2.5 + 125	5 + 250	62.5	125	250	1.25 + 62.5	2.5 + 125	5 + 250
	mg/mL			*µ*g/mL			mg/mL + *µ*g/mL			*µ*g/mL			mg/mL + *µ*g/mL		

*Escherichia coli* ATCC 25922	13	15	18	22	25	30	16	19	21	23	25	28	16	20	23
*Bacillus cereus* ATCC 10702	17	20	23	25	28	30	19	20	24	24	27	30	15	17	21
*Pseudomonas aeruginosa* ATCC 19582	16	18	21	25	28	30	20	22	26	22	25	28	20	23	26
*Serratia marcescens* ATCC 9986	0	14	18	0	0	0	16	20	24	23	25	28	19	24	26
*Enterococcus faecalis* KZN	17	20	23	0	0	0	0	0	0	0	0	0	0	0	0
*Staphylococcus aureus* OK1	17	19	21	21	25	28	17	20	23	18	22	27	22	26	28
*Salmonella typhi* ATCC 13311	25	29	32	30	32	35	23	27	30	28	30	32	29	32	34
*Micrococcus luteus *	14	16	19	29	30	35	20	24	27	22	25	28	20	24	26
*Proteus vulgaris* CSIR 0030	18	21	23	25	28	30	25	26	28	23	26	28	25	27	29
*Shigella flexneri* KZN	20	22	24	25	27	29	25	26	28	19	23	25	23	25	27

AMA: acetone extract of *A. mearnsii*; Ery: erythromycin; Tet: tetracycline; Met: metronidazole; Cip: ciprofloxacin; Nal: nalidixic acid; Chl: chloramphenicol; Kan: kanamycin.

**Table 2:** Antibacterial effects of acetone stem bark extract of *A. mearnsii* and the different first-line antibiotics.

Tested bacterial isolates	Minimum inhibitory concentrations (MIC)								
	AMA	Ery	Tet	Met	Amx	Cip	Nal	Chl	Kan
	mg/mL	*µ*g/mL							
*Escherichia coli* ATCC 25922	0.625	0.391R	0.976S	31.250R	3.906S	0.039S	1.953S	3.906S	125R
*Bacillus cereus* ATCC 10702	0.156	0.098S	0.244S	31.250R	7.813R	0.078S	15.625S	3.906S	125R
*Pseudomonas aeruginosa* ATCC 19582	0.156	0.195S	0.488S	15.625R	3.906S	0.156S	7.813S	3.906S	31.25R
*Serratia marcescens* ATCC 9986	0.625	3.125R	15.625R	31.250R	31.25R	0.078S	1.953S	0.977S	1.953S
*Enterococcus faecalis* KZN	0.625	12.500R	15.625R	62.500R	0.977S	0.313S	62.500R	31.250R	500R
*Staphylococcus aureus*_ OK1_	0.078	0.195S	0.976S	15.625R	0.977S	0.078S	62.500R	7.813R	15.625R
*Salmonella typhi* ATCC 13311	0.313	6.250R	0.244S	31.250R	0.977S	0.0195S	7.813S	3.906S	15.625R
*Micrococcus luteus *	0.039	0.391R	31.250R	31.250R	0.488S	1.250R	62.500R	1.953S	250R
*Proteus vulgaris* CSIR 0030	0.156	0.048S	0.488S	62.500R	0.244S	0.0195S	62.500R	31.250R	31.25R
*Shigella flexneri* KZN	0.078	0.195S	0.488S	62.500R	250.000R	0.039S	62.500R	7.813	15.625R

S: sensitive; R: resistant; AMA: acetone extract of *A. mearnsii*; Ery: erythromycin; Tet: tetracycline; Met: metronidazole; Cip: ciprofloxacin; Nal: nalidixic acid; Chl: chloramphenicol; Kan: kanamycin.

**Table 3:** Fractional inhibitory concentrations of acetone stem bark extract of *A. mearnsii* in combination with the different antibiotics.

Tested bacterial isolates	Fractional inhibitory concentrations (FIC)							
	AMA + Ery	FICI	AMA + Tet	FICI	AMA + Met	FICI	AMA + Amx	FICI
	mg/mL + *µ*g/mL		mg/mL + *µ*g/mL		mg/mL + *µ*g/mL		mg/mL + *µ*g/mL	
*Escherichia coli* ATCC 25922	0.625/6.25	17.00Ant	0.0098/0.244	0.266 Syn	0.313/15.625	1.00 Add	0.156/7.813	1.25 Ind
*Bacillus cereus* ATCC 10702	0.0098/0.098	1.63 Ind	0.00122/0.061	0.258 Syn	0.156/7.813	1.25 Ind	0.078/3.906	1.000 Add
*Pseudomonas aeruginosa* ATCC 19582	0.0049/0.049	0.06 Syn	0.00122/0.061	0.133 Syn	0.625/31.250	6.00 Ant	0.039/1.953	0.750 Add
*Serratia marcescens* ATCC 9986	0.625/6.25	3.00 Ind	0.0781/3.906	0.37 Syn	0.625/31.250	2.00 Ind	0.156/7.813	0.500 Syn
*Enterococcus faecalis* KZN	0.156/1.563	0.38 Syn	0.313/15.625	1.5 Ind	0.625/31.250	1.50 Ind	0.0049/0.244	0.258 Syn
*Staphylococcus aureus*_ OK1_	0.0049/0.049	0.31 Syn	0.0098/0.244	0.375 Syn	0.625/31.250	10.00 Ant	0.0049/0.244	0.312 Syn
*Salmonella typhi* ATCC 13311	0.1563/1.953	0.81Add	0.00122/0.061	0.254 Syn	0.625/31.250	3.00 Ind	0.0049/0.244	0.265 Syn
*Micrococcus luteus *	0.0391/0.391	2.00 Ind	0.0781/3.906	2.128 Ind	0.313/15.625	10.00 Ant	0.0024/0.122	0.312 Syn
*Proteus vulgaris* CSIR 0030	≤0.0003/0.003	0.08 Syn	0.0049/0.122	0.281 syn	0.078/3.906	0.50 Syn	0.006/0.030	0.165 Syn
*Shigella flexneri* KZN	0.0098/0.098	0.38 Syn	0.156/7.813	18.01 Ant	0.313/15.625	4.25 Ant	0.0391/1.953	0.509 Add

Tested bacterial isolates	Fractional inhibitory concentrations (FIC)							
	AMA + Cip	FICI	AMA + Nal	FICI	AMA + Chl	FICI	AMA + Kan	FICI
	mg/mL + *µ*g/mL		mg/mL + *µ*g/mL		mg/mL + *µ*g/mL		mg/mL + *µ*g/mL	

*Escherichia coli* ATCC 25922	0.0098/0.0098	0.266 Syn	0.078/3.906	2.125 Ind	0.0391/1.953	0.563 Add	0.156/7.813	0.313 Syn
*Bacillus cereus* ATCC 10702	0.0781/0.0781	1.500 Ind	0.313/15.625	3.000 Ind	0.0195/0.977	0.375 Syn	0.078/3.906	0.531 Add
*Pseudomonas aeruginosa* ATCC 19582	0.0781/0.0781	1.000 Add	0.156/7.813	2.000 Ind	0.0195/0.977	0.375 Syn	0.0391/1.953	0.312 Syn
*Serratia marcescens* ATCC 9986	0.0098/0.0098	0.250 Syn	0.039/1.953	1.062 Ind	0.0098/0.488	0.512 Add	0.078/3.906	2.125 Ind
*Enterococcus faecalis* KZN	1.25/1.25	6.000 Ant	0.625/31.250	1.500 Ind	0.0781/3.906	0.250 Syn	1.250/62.500	2.125 Ind
*Staphylococcus aureus*_ OK1_	0.0098/0.0098	0.251 Syn	0.156/7.813	2.125 Ind	0.0391/1.953	0.750 Add	0.313/15.625	5.000 Ant
*Salmonella typhi* ATCC 13311	0.0049/0.0049	0.27 Syn	0.039/1.953	0.37 Syn	0.0195/0.977	0.44 Syn	0.0097/0.488	0.062 Syn
*Micrococcus luteus *	0.3125/0.3125	8.250 Ant	0.156/7.813	4.125 Ant	0.0391/1.953	2.000 Ind	0.625/31.500	16.13 Ant
*Proteus vulgaris* CSIR 0030	0.0049/0.0049	0.283 Syn	0.078/3.906	0.562 Add	0.0781/3.906	0.626 Add	0.0391/1.953	0.312 Syn
*Shigella flexneri* KZN	0.0098/0.0098	0.377 Syn	0.313/15.625	4.250 Ant	0.0391/1.953	0.750 Add	0.313/15.625	5.000 Ant

AMA: acetone extract of *A. mearnsii*; Ery: erythromycin; Tet: tetracycline; Met: metronidazole; Cip: ciprofloxacin; Nal: nalidixic acid; Chl: chloramphenicol; Kan: kanamycin; FICI: fractional inhibitory concentration indices.
